# Cross-talk between the infant/maternal gut microbiota and the endocrine system: a promising topic of research

**DOI:** 10.20517/mrr.2021.14

**Published:** 2022-03-31

**Authors:** Francesca Turroni, Sonia Mirjam Rizzo, Marco Ventura, Sergio Bernasconi

**Affiliations:** ^1^Laboratory of Probiogenomics, Department of Chemistry, Life Sciences, and Environmental Sustainability, University of Parma, Parma 43124, Italy;; ^2^Microbiome Research Hub, University of Parma, Parma 43124, Italy.

**Keywords:** Infants, gut microbiota, endocrine system, thyroid, hormones

## Abstract

The infant gut microbiota is the set of microorganisms colonizing the baby’s intestine. This complex ecosystem appears to be related to various physiological conditions of the host and it has also been shown to act as one of the most crucial determinants of infant’s health. Furthermore, the mother’s endocrine system, through its hormones, can have an effect on the composition of the newborn’s gut microbiota. In this perspective, we summarize the recent state of the art on the intricate relationships involving the intestinal microbiota and the endocrine system of mother/baby to underline the need to study the molecular mechanisms that appear to be involved.

## THE GUT MICROBIOTA ESTABLISHMENT

The gut microbiota includes autochthonous and / or allochthonous microorganisms colonizing the intestinal tract. The impact of a correct balance of the gut microbiota on the health and entire physiology of the host from infancy is widely accepted by the scientific community. The establishment of this complex microbiota occurs immediately after birth and is influenced by different factors, such as gestational age, mode of delivery, type of feeding^[1,2]^, antibiotic exposure^[3-5]^, host genetics, and the environment^[6]^. Other factors related to the mother’s health conditions may also influence the microbiota of the fetus. For example, maternal nutrition during pregnancy, which directly modulates her microbiota, may also impact the initial microbial establishment of the newborn^[7]^. Maternal diet seems to similarly shape the composition and diversity of breast milk microbiota, especially for macronutrients and soluble/insoluble fibers, plant/animal proteins, and polyphenol composition^[8]^. Furthermore, maternal dietary interventions, i.e., with vitamin D and polyunsaturated fatty acids, play an essential role in early bacterial colonization in various body districts differently from the gut, such as the lung, by modulating the pulmonary microbiota and thus reducing the incidence of asthma and wheezing in offspring^[9]^. This supports the fact that the gut microbiota of the mother can be a vehicle for the baby’s health. Therefore, the first months of life are essential in favoring the bacterial colonization of the infant’s gut to the detriment of pathogens. This intestinal microbial population develops rapidly after birth up to three years of age when the microbiota reaches certain stability and complexity^[10]^. The establishment of a balanced gut microbiota during infancy represents the foundation of health with long-lasting effects in adulthood. For example, in addition to the maternal overweight/obesity condition that can affect some of the infant gut microbiota taxa, associated with subsequent body mass index, the gut microbiota of early childhood could influence the development of obesity in the postnatal life^[11]^. Moreover, the maternal gut environment during pregnancy contributes in a fundamental way to the metabolic programming of the neonate to prevent metabolic syndrome via neural systems. The maternal gut microbiota confers resistance to obesity in offspring via the free fatty acids receptor stimulation, representing signaling molecules between the gut microbiota and extraintestinal organs^[12]^.

Another factor highly correlated with the correct establishment of the infant intestinal microbiota is the mother’s immune system and the newborn itself. The transplacental route is the normal avenue through which mothers give natural passive immunization to their babies^[13,14]^. It seems that the immune status during pregnancy, in terms of immunoglobulins and cytokines levels, can also influence the immune status of the newborn^[15]^. After birth, the immune system develops in early childhood, mainly through interactions with the gut microbiota^[16]^. Other important contributors in the development of the immune system at the early stages of life are represented by specific ingredients of the human milk as the human milk oligosaccharides (HMO). These metabolic products can influence the immune system development, specifically reducing mucosal and systemic inflammation^[17]^. In this context, some species belonging to the genus *Bifidobacterium *resulted in being specifically enhanced by HMOs, which ultimately are considered to be directly involved in immunoregulation during the first months of life^[17]^. These early-life immune–microbial interactions appear to affect the risk of allergies, asthma, and other inflammatory diseases, showing that the imprinting of early immunity can influence later health through various mechanisms that are still not fully understood today^[17]^. The microbiota changes, especially after the introduction of solid foods, evolves during adulthood, and then undergoes a decrease in microbial richness observed in aging populations, linked to various factors (e.g., changed lifestyle habits, reduced food diversity, and introduction of drugs)^[18]^. The gut microbiota has various effects on the gut environment that also impact distant organs and pathways, such as the nervous^[19,20]^ and endocrine systems^[21]^. These effects are very important given the enormous implications on the individual’s health, but they are still largely unknown. This discourse enters a broader and more fascinating context concerning the different cross-kingdom cell-to-cell signaling involving small molecules^[22,23]^. Nevertheless, there are several questions to be answered, such as how do hormone-like chemicals produced by intestinal bacteria affect host signaling? How do the mother’s hormones affect the infant’s microbiota composition? What are the molecular tools that should be used to disclose this topic?

This perspective summarizes the scientific evidence currently existing on the molecular mechanisms involved in the interactions between the intestinal microbiota and the endocrine system, which unfortunately are still very limited, thus making this topic a very promising area of research for the next years.

## INTERACTION OF THE GUT MICROBIOTA AND THE HUMAN ENDOCRINE SYSTEM 

**Figure 1:**
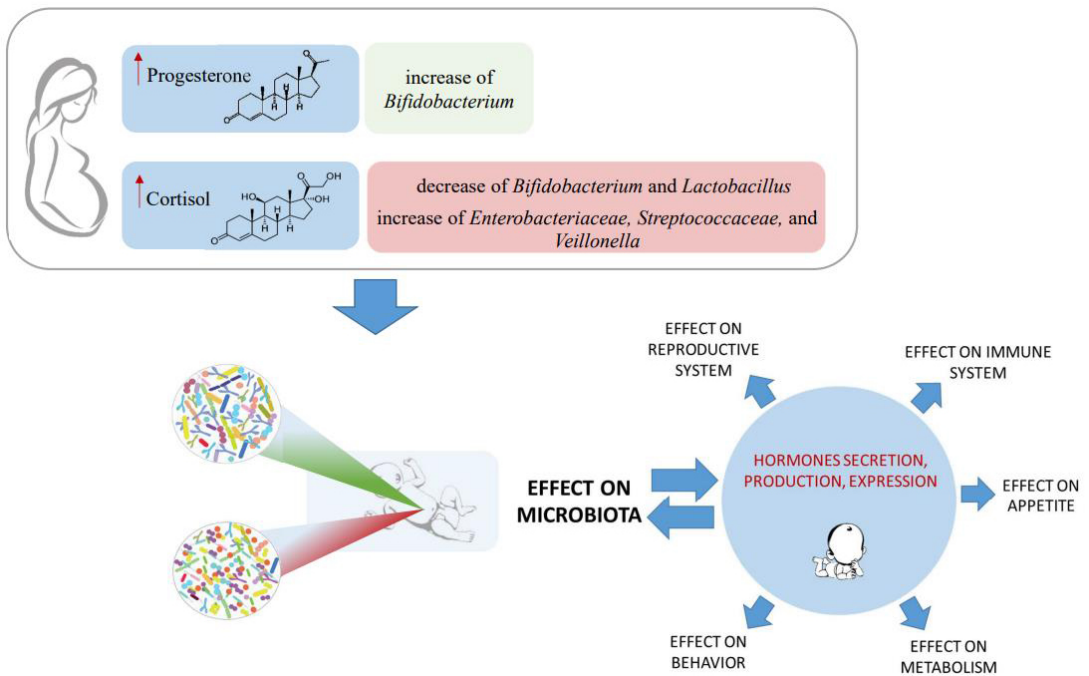
Schematic representation of the relationships between the infant gut microbiota and the endocrine system of newborn/mother. There is a reciprocal interaction between the infant gut microbiota and hormones’ secretion, production, and expression. Additionally, a change in the number of hormones in the pregnant mother impacts her gut microbiota, affecting the baby’s microbiota.

The first indication concerning the existence of a cross-talk between the microbiota and the endocrine system dates back to 1992^[24]^. Since then, many reports have been published on this topic^[21,25,26]^. Nowadays, it is known that, in some cases, specific changes in hormone levels are somehow related to the presence and composition of the gut microbiota [Table 1]. Despite this, a large part of the research effort has been placed on attempting to elucidate the specific molecular mechanisms of this interaction, which are far from being fully understood. In this context, it has been shown that the microbiota is involved in both production and secretion and itself is modulated in response to hormones. However, the precise molecular mechanisms of each microbiota-hormone signaling have not yet been clarified. Furthermore, the human microbiota and endocrine cross-talks affect a variety of host responses such as behavior, metabolism, appetite, immune system, and reproduction, emphasizing the complexity of this fascinating topic [Figure 1].

**Table 1:** Currently published studies focusing on the hormone-microbiota relationships

**Hormone category**	**Hormones**	**Ref.**
Breast milk-related hormones	Leptin, adiponectin, insulin, ghrelin, obestatin, apelin, resistin, irisin, copeptin, nesfatin, GLP-1, IGF-1, melatonin	[27-29]
Reproductive related hormones	Progesterone	[30-33,41,42]
	Estrogen	
	Androgen	
Stress-related hormones	CortisolIodothyronines	[52,58,59,87,88,92,99-101]

GLP: Glucagon-like peptide; IGF: insulin-like growth factor.

A significant example of this interaction is represented by the influence of changes in the gut microbiota compositions in response to various appetite-related hormones (e.g., leptin, insulin, and ghrelin), which play key roles in modulating brain behavior and function through the humoral or neural pathway^[27]^. In postnatal life and throughout adulthood, leptin deficiency causes elevated endoplasmic reticulum stress in various metabolically relevant tissues, particularly in the hypothalamic circuits^[28]^. Moreover, gut dysbiosis, i.e., a disruption of the natural gut microbiota homeostasis, improves leptin sensitivity and can contribute to the high level of leptin by epigenetically modulating its expression in obese condition^[29]^.

Only recently, it emerged that the microbiota plays an important role in the endocrine system, especially in the reproductive system of woman, interacting with estrogen^[30,31]^, androgen^[32,33]^, insulin^[34,35]^, and other hormones^[21,25]^. Moreover, an imbalanced gut microbiota composition has led to various diseases and disorders, such as adverse pregnancy outcomes, pregnancy complications, polycystic ovary syndrome (PCOS), endometriosis, and cancer^[36]^. However, the actual molecular mechanisms underlying this phenomenon have not yet been clarified.

Nutritional and hormonal disruptions that occur early in human life can promote an alteration of the individual’s metabolic programming later in life and modify the gut microbiota composition^[37]^. Indeed, changes in the gut microbiota composition, in response to alterations of sex hormones, may trigger the gene expression response, via miRNAs, in the host^[37^].

Early evidence shows that some hormones appear to have a direct effect on specific bacterial taxa, as steroid hormones have been shown to increase the growth of *Prevotella intermedia* and *Prevotella melaninogenica* in the oral cavity^[38]^.

Furthermore, gut bacteria produce different metabolites that could act as signaling molecules to several cell types within the mucosa. On the other hand, enteroendocrine cells produce and secrete several hormones, which have regulatory roles in key metabolic processes such as insulin sensitivity, glucose tolerance, fat accumulation, and appetite.

Another important sign underlying the existence of a direct cross-talk between hormones and microbiota is represented by the fact that the gut microbiota, as well as the vaginal, oral, and skin microbiota, undergoes changes during different pregnant trimesters, thus suggesting that sex hormones could be responsible for these modifications^[39,40]^. It has been shown that reproductive hormones, specifically progesterone, which is the main hormone produced during pregnancy, impact the gut microbiota shifts during pregnancy and lactation in Phayre’s leaf monkeys^[41]^.

Interestingly, *in vitro *and* in vivo *experiments showed that progesterone promotes the growth of key gut microbiota members such as bifidobacteria during late pregnancy^[42] ^[Figure 1]. Although the precise mechanism has not yet been clarified, it has been proposed that it may depend on the presence of the hydroxysteroid dehydrogenase (HDS) enzyme in bifidobacteria^[43] ^or on a specific unknown regulator stimulated by progesterone^[42]^. The increase of bifidobacteria load during late pregnancy might not only be helpful for pregnancy (i.e., reduction in the incidence of pre-term births^[44]^) but also reflect an evolutionary process of preparation for birth and feeding time^[42]^. In fact, bifidobacteria represent the dominant gut microbiota members in the early stages of life that are vertically transmitted from the mother to the newborn^[45,46]^.

Recently, in both human and animal studies, an intriguing relationship between the composition of the gut microbiota and the sex of the individual has been shown. Changes in gut microbiota communities due to sex are linked to the interaction of sex hormones with the immune system^[47,48]^. In addition, it has been proposed that, since bile acids are different in composition between men and women, and since these chemical compounds exploit a key role in the gut microbiota composition, this could represent a possible mechanism explaining that sexual differences influence the gut microbiota composition^[49]^.

## THE INTERACTION OF MICROBIOTA AND NEUROENDOCRINE SYSTEM

In the last decades, strong bidirectional connection and influence between the gut microbiota and the endocrine, immune, and neural systems have been demonstrated^[50,51]^.

**Figure 2:**
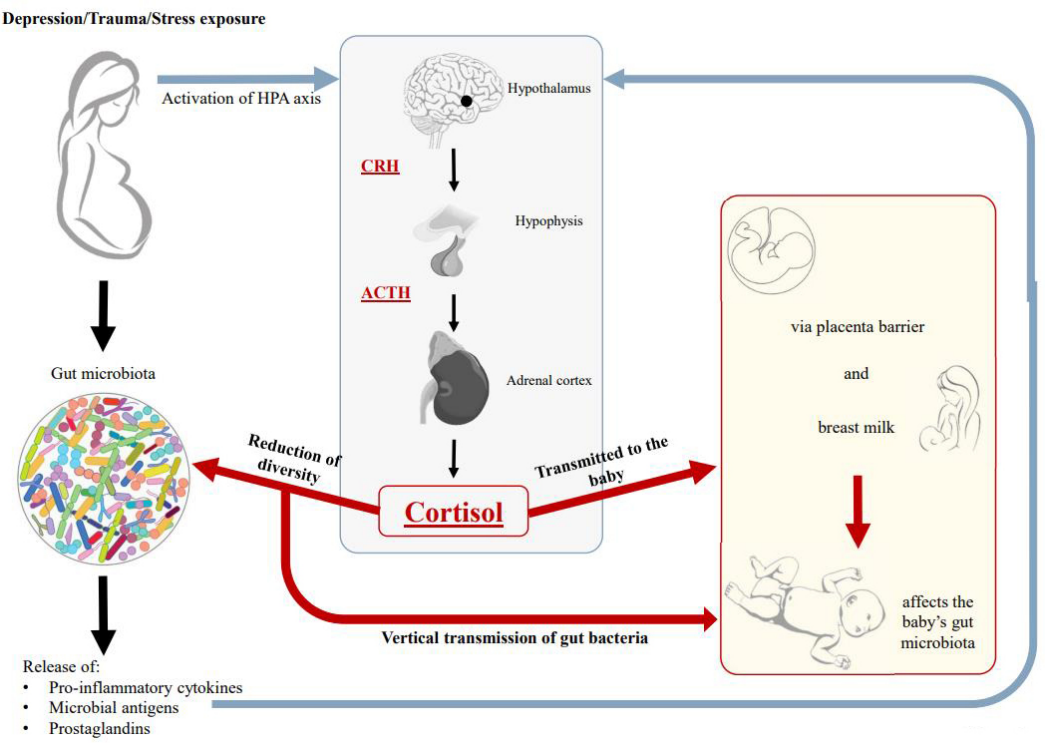
Schematic representation of the relationship between the HPA (hypothalamus-hypophysis-adrenal cortex) axis and the gut microbiota of both mother and child. Traumatic and stressful events during pregnancy lead to a hyper-activation of the hypothalamus with the release of corticotrophin-releasing hormone(CRH) that induces the production of the adrenocorticotrophic hormone (ACTH) secreted by the hypophysis. In addition, ACTH causes the release of cortisol from the adrenal cortex. Cortisol is a glucocorticoid that directly and indirectly changes the composition of the child’s gut microbiota. This hormone is transmitted to the baby via breastfeeding and likewise from the mother’s bloodstream through the placenta barrier. Moreover, cortisol affects the composition of the mother’s gut microbiota, which is then passed on to the child through vertical transmission.

The so-called microbiota gut-brain axis communication occurs primarily with the interaction of the intestinal microbiota and the hypothalamic-pituitary-adrenal (HPA) pathway^[52,53] ^[Figure 2]. The HPA axis is a neuroendocrine pathway constituted by the hypothalamus, the hypophysis, and the adrenal glands. The activation of this axis, given by stress exposure, leads to an adaptation to environmental requests through the activation of corticotrophin-releasing hormone by the hypothalamus, which induces the production of the adrenocorticotrophic hormone (ACTH) secreted by the hypophysis. ACTH in turn leads to the release of glucocorticoids, including cortisol, from the adrenal cortex^[54]^. This steroid hormone regulates a wide range of processes throughout the body; for example, hyper-secreted cortisol induces an increase in visceral adiposity, decreases lean mass (muscle and bone), and suppresses osteoblastic activity^[55]^.

The intestinal microbiota activates this neuroendocrine pathway, affecting the secretion of cortisol and the physiological response to stressors because of the release of mediators such as pro-inflammatory cytokines, microbial antigens, and prostaglandins, which are able to cross the blood–brain barrier^[54]^. In the case of intestinal dysbiosis, the microbiota may induce a constant hyperactivity of the HPA axis, leading to deleterious effects on the organism. At the same time, a condition of acute or chronic stress, together with an increment of cortisol levels, can increase the gut permeability, inducing autoimmunity and reducing the diversity of the gut microbiota^[52-54]^. Many studies have suggested that there is a strong link between changes in the gut microbiota composition and the outbreak of psychological outcomes such as anxiety-like symptoms and depression already at a young age^[56]^. Experimental results in mice suggest that a dysbiosis of the gut microbiota could turn into psychological disrupted behavior, promoting the establishment of depression^[56,57]^. The molecular pathway underlying this relationship is not yet understood, but a strong indication supporting this hypothesis is that the early changes in the composition of the gut microbiota can affect different aspects of brain function and behavior later in adulthood through the HPA axis and stress response^[58]^. As mentioned above, a mother’s intestinal health during pregnancy contributes in a fundamental way to the metabolic programming of the newborn through the nervous system^[12]^. The mother’s well-being positively affects the infant’s health in the same way conditions of physical or mental illness can adversely influence the developing fetus^[59-61]^. Much evidence indicates that women who suffered from stressful events such as depression or exposure to trauma during pregnancy (e.g., food insecurity, low social support, and socioeconomic hardship] display dysregulation of the HPA pathway^[59]^). As a main consequence, an increase of circulating cortisol, a steroid hormone that regulates a wide range of processes throughout the body, has been detected in salivary samples of pregnant women^[59]^. Moreover, infants of mothers who experienced traumatic events or who presented elevated levels of cortisol during pregnancy had a lower relative abundance of *Lactobacillus* and *Bifidobacterium. *In addition, they displayed significantly higher relative abundances in other microbial groups including potentially pathogenic bacteria such as *Enterobacteriaceae, Streptococcaceae,* and *Veillonella* in their gut microbiota^[59,61]^. Three main mechanisms have been proposed to explain how maternal cortisol affects the infant gut microbiota. First, a high cortisol level interferes with the mother’s gut microbiota composition, influencing the transmission of intestinal bacteria from mother to infant. Second, cortisol can also cross the placenta barrier, directly increasing the circulating level of this hormone in the fetus with a dysregulation of the HPA axis. Third, cortisol is transmitted through breast milk, ultimately shaping the infant gut microbiota since it is widely accepted that components of breast milk, such as HMOs, largely influence infants’ gut microbiota^[10]^[Figure 2].

Alteration of glucocorticoid levels during the prenatal period as well as during infancy and early childhood development could have long-term effects^[62]^. The activity of the HPA axis has been estimated by measuring cortisol levels in salivary samples taken before and after a mild physical stress (heel stick) from infants around one month of age. Together with the information of the microbiota composition of fecal samples taken from the infants, these data suggest that certain bacteria, such as *Staphylococcus*, *Prevotella*, and other microbial genera belonging to the order *Clostridiales*, may be associated with increased cortisol reactivity following physical stress^[58]^. The increase in cortisol level can produce several consequences, such as immune stimulation production of bile acid in the liver^[59,63]^, affecting the intestinal motility, and alterations in the integrity of the epithelial barrier^[64]^, which could, in turn, affect the gut microbiota. These dysregulations would induce a pro-inflammatory state with neurological relapses^[64]^. Consequently, altered basal and reactive cortisol patterns can impact the development of the child’s emotional and behavioral regulation systems, later causing social problems and psychopathology^[62]^. Other studies suggest that the restoration of healthy gut microbiota with the administration of probiotics belonging to the *Lactobacillus *and *Bifidobacterium* genera can improve anxiety-like symptoms resulting from HPA axis hyperactivity^[58,65]^. Fecal microbiota transplantation administered to neonates born by caesarean section represents a promising frontier of study in imprinting the correct development of the intestinal microbiota^[66]^. Another possible intervention strategy is represented by probiotics that specifically target the mediated functions and behaviors of the central nervous system, called psychobiotics^[67]^. These products act through immune, humoral, neural, and metabolic pathways to not only improve intestinal function but also produce an antidepressant and anxiolytic capacity^[68]^. Nevertheless, further in-depth studies are required to explore the relationship existing between the infant gut microbiota and the HPA axis, which might be crucial to develop novel therapies/strategies to reduce unhealthy stress responses by interfering with the infant microbiota.

## BREAST MILK AND HORMONES

**Figure 3:**
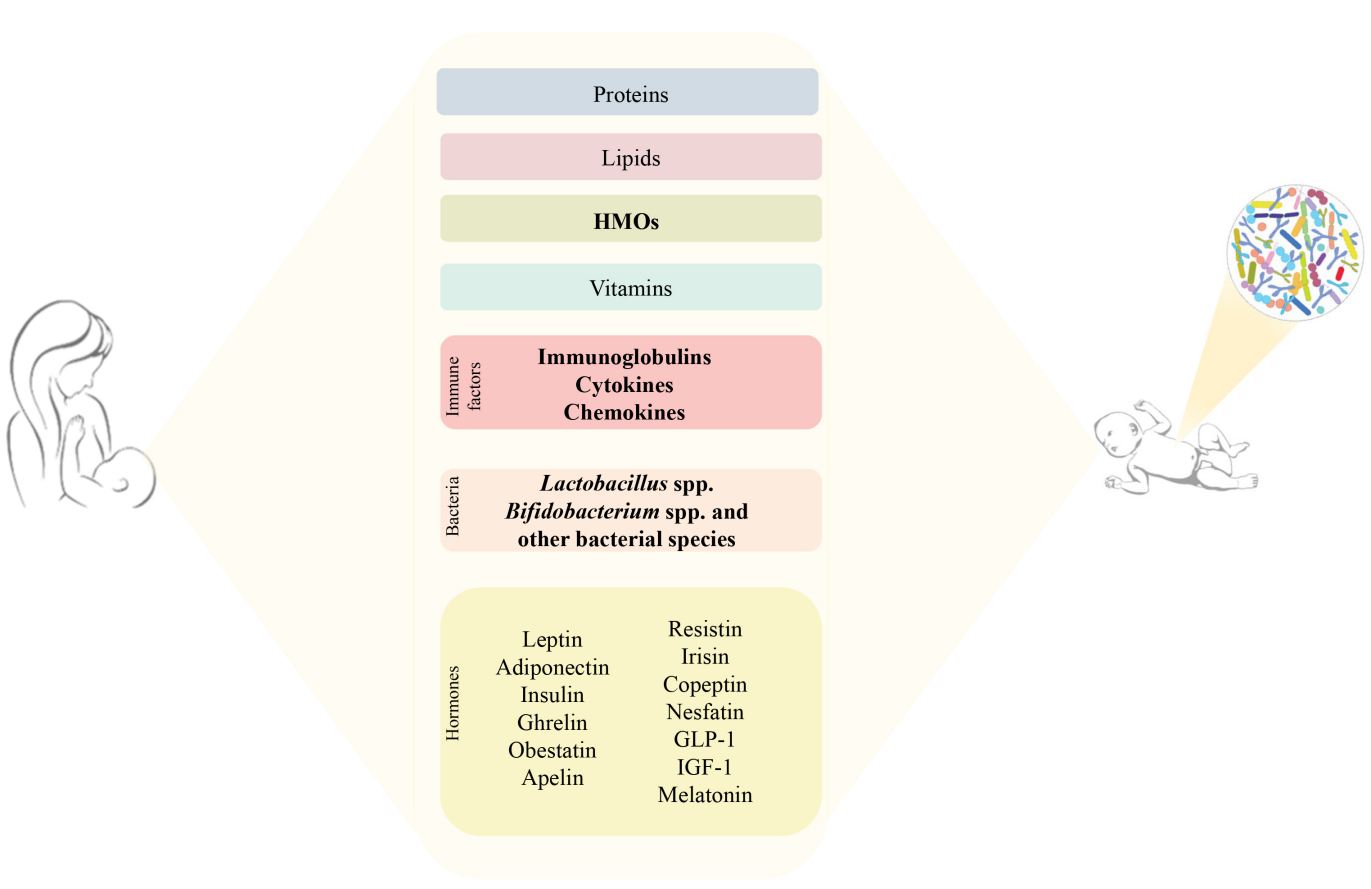
Schematic representation of the human milk breast components. Breast milk is the vehicle for essential compounds, not only nutritional, listed in the figure. The components that directly influence the gut microbiota composition are highlighted in bold.

Maternal milk contains different nutrients that are changing in their nature and amounts over the time of breastfeeding due to the mother’s hormonal, physiological, and neuroendocrine mechanisms^[69,70]^, as well as being influenced by genetic and environmental factors and eating habits^[71] ^[Figure 3]. Besides the fundamental constituents, namely proteins, lipids, carbohydrates, and vitamins^[71,72]^, maternal milk contains bioactive molecules that shape and develop the newborn’s gut microbiota and immunological system, such as immunoglobulins, cytokines, and chemokines^[70,72]^. It is also a source of bacteria, mainly belonging to the genus *Bifidobacterium*, which are important for establishing the infant gut microbiota^[10]^. Together, all these chemical compounds predispose the newborn’s optimal physiological and neurological development^[73]^. In addition, the importance of other molecules, such as hormones including leptin, adiponectin, insulin, ghrelin, obestatin, apelin, resistin, irisin, copeptin, nesfatin, glucagon-like peptide-1 (GLP-1), and insulin-like growth factor-1 (IGF-1), has emerged, and their involvement with the baby’s growth and development has intrigued the scientific community^[69] ^[Table 2]. These hormones immediately control the newborn’s sense of satiety, and, in the later stages of life, they influence the energy balance^[69]^. Breastfed infants display lower circulating insulin and IGF-I levels than babies who are not fed with human milk^[74]^. In fact, these compounds are not found in artificial formula, suggesting that breastfed infants are less likely to gain weight than those bottle-fed^[74]^. The strong correlation between the health of the mother and that of the child is also highlighted by the fact that obese mothers have a higher concentration of insulin, leptin, and pro-inflammatory fatty acids in breast milk than normal-weight mothers^[69]^. Furthermore, in this same category of mothers, breast milk is characterized by less microbial diversity and a reduction in *Bifidobacterium* spp. levels and cytokine content^[75-77]^. Another milk-related hormone influencing baby behavior is melatonin, produced primarily in the pineal gland. Following a circadian cycle, this hormone reaches breast milk, where it plays a role in regulating sleep and seems to have a possible involvement in gut-brain communications^[78]^. Melatonin displays a wide range of biological functions such as antioxidants, anti-inflammatory, antinociceptive, immune regulators, and maintaining gut-barrier integrity^[78]^. 

**Table 2:** Functions and effects of the hormones contained in human breast milk

**Hormone**	**Function**	**Effects on newborns **	**Ref.**
Leptin	Suppress the appetite	Early sense of satiety	[104]
Adiponectin	Improves glucose metabolism by increasing the insulin sensitivity	Higher adiponectin concentration is associated with more significant neonatal weight gain	[105]
Insulin	Energetic homeostasis	Decreased lean mass	[106]
Ghrelin	Intestinal motility, energy homeostasis	Its concentration is positively correlated with the weight and size of the neonate	[106]
Obestatin	Associated with the bodyweight decrease	No effects reported yet	[107]
			
Resistin	Inhibits adipocyte differentiation	No effects reported	[108]
Irisin	Transforming the white adipose tissue in brown adipose tissue	Stimulate thermogenesis in white adipose tissue	[109]
Copeptin	Regulation of water excretion	No effects reported yet	[110]
Apelin	Fasting plasma levels positively correlate with BMI	No effects reported yet	[111]
NefastinGLP-1IGF-1	Regulation of appetiteInduce satietyMay stimulate weight gain	No effects reported yetNo effect reported yetMajor rate of growth of the infant during the first month of life	[112][113][114]
Cortisol	Increase the blood glucose level through gluconeogenesis, suppress the immune system	May support to program metabolic functioning and childhood obesity risk.Have been associated with infant temperament	[115]
Melatonin	The antioxidant, anti-inflammatory, antinociceptive, immune regulator, and maintaining gut-barrier integrity	Help to sleep May modulate gut microbiota	[78,116]

GLP: Glucagon-like peptide; IGF: insulin-like growth factor.

Melatonin appeared to increase the relative abundance of gut Actinobacteria in a suckling pigs^[79]^. In addition, it influences the swarming activity of the intestinal bacteria *Enterobacter aerogenes *^[80]^, suggesting a possible inter-kingdom communication mechanism. This first result underlines that the field of intestinal hormone–microbiota relationships is very complex, vast, and still largely unknown, especially for the molecular mechanisms involved in the interaction between microbes and maternal hormones. Therefore, a very fascinating and promising area of research will be to dissect the impact of all the human breast milk hormones [Table 2] on the various members of the infant gut microbiota and how each hormone could affect the transcriptomes of the different infant gut microbes. 

## THYROID HORMONES AND GUT MICROBIOTA INVOLVEMENT 

During growth, thyroid hormones play a crucial role in some physiological processes; in fact, these are regulators of growth as well as in the myelination process of the nervous system, metabolism, and other organ functions^[81]^. Consequently, the normal proper functioning of the thyroid gland contributes to maintaining the internal balance of the whole organism^[82]^. In this context, adequate diagnosis and management of thyroid disease during pregnancy are important for maternal and fetal health^[83]^.

In recent years, particular attention has been placed on the possible relationship between microbiota and thyroid function (thyroid-gut axis) ^[84]^. Moreover, experimental data highlight that intestinal microbiota transplantation from hypothyroid mice to healthy mice leads to decreased thyroid function in the latter^[85]^, suggesting a clear association between thyroid function and intestinal bacteria. However, we are still far from understanding the real mechanisms of the connection between microbiota and thyroid function in humans, especially in pregnant women, and the possible consequences in clinical practice. Nevertheless, some data from animal model-based studies allow us to suggest various potential roles, among them the action of the microbiota on the metabolism of iodothyronines^[84]^ and the absorption of micronutrients, which are essential for normal thyroid function (iodine, iron, copper, zinc, and, above all, selenium)^[86]^. Furthermore, several studies in humans have shown, albeit unevenly, the presence of dysbiosis in *Helicobacter pylori *(HP)and Hashimoto’s thyroiditis (HT) patients^[87]^. In the latter case, a correlation between the abundance of selected types of bacteria and diagnostics parameters has been observed, connected with autoimmune thyroiditis, such as antibodies to the thyroid gland peroxidase (anti-TPO) and thyroglobulin (anti-TG)^[88]^. Moreover, in subjects affected by hyperthyroidism, there are differences in the microbial composition of the gut microbiota; specifically, there is a high Firmicutes/Bacteroidetes ratio^[88]^, which, interestingly, was proposed but not accepted as a biomarker of obesity^[89]^.

Studies on the relationship between microbiota and the bioactivity of the drugs currently used to achieve a normal functional level in thyroid diseases are of particular interest. Bioactivity deserves particular interest because it can explain the high inter-individual variability of the therapeutical response, such as l-thyroxine (LT-4), which is known to be the drug of choice in the treatment of hypothyroidism. In addition, this is a fascinating new area of research called pharmacomicrobiomics, whose purpose is to demonstrate how the microbiota can, directly or indirectly, modify the efficacy of many drugs, in addition to antibiotics whose effects on the gut microbiota are known^[90]^. On the one hand, it is well known that synthetic drugs used to treat HP or Graves’ disease (GD) patients may modify the gut microbiota *in vivo* during disease treatment. For example, Yao et al. showed that the gut microbiota of hypothyroid patients, treated with L-thyroxine, varied among individuals dependently by L-thyroxine dosages^[91]^. Similarly, Sun et al. verified that methimazole and propylthiouracil, which represent the first line of hyperthyroidism treatment, altered the gut microbiota composition^[92]^. Moreover, the gut microbiota can also metabolize some synthetic drugs, modulate the expression of some host cytochrome enzymes that can metabolize drugs, and directly produce some enzymes that participate in drug absorption, activation, and inactivation^[92]^.

L-thyroxin is absorbed in the small intestine and metabolized by peripheral deiodination, but it also undergoes a conjugation in the liver. Glucuronidation of thyroxine is a major metabolic pathway facilitating its excretion through biliary flow. In humans and rats, it has been demonstrated that many conjugated iodothyronines can be hydrolyzed by the gut microbiota^[93]^, thus showing that intestinal microbiota can mediate this very complex metabolism. In fact, it has been shown that some microorganisms display a glucuronidase activity, and therefore they might metabolize thyroid hormones^[94-96]^. The deconjugation by gut bacteria prevents the fecal loss of thyroxine, thus enabling the enterohepatic recycling of the hormone. As underlined by Virili et al., there are insufficient data to discern its net contribution to the whole thyroid homeostasis^[97^].

Moreover, gut microbiota may significantly modify the intestinal absorption surface by regulating the expression of tight junctions, affecting intestinal permeability, the shape of enterocytes, and the composition of the mucus layer^[97]^. Moreover, it can be assumed that the gut microbiota composition may influence the efficacy of oral LT-4 by direct binding to bacteria, as has been displayed for *E. coli*^[98]^. The relationship between LT-4 therapy and microbiota needs to be further investigated. In this context, a randomized study has shown a better response in normalization of stimulating hormones (TSH)and the ratio FT3/TSH and a lower dosage of LT-4 in hypothyroid patients treated with LT-4 and symbiotic as compared to patients treated with LT-4 e alone^[99]^.

In addition to the possible modulation of the pharmacological action, many studies have focused on the possible role of the microbiota in the pathogenesis of HT and Graves’ disease, which are two of the most frequent thyroid diseases of which the autoimmune origin is recognized. Furthermore, it is known that there is an interaction between microbiota and immunity, both innate and adaptive, and that in various autoimmune diseases (type 1 diabetes, rheumatoid arthritis, celiac disease, Sjogren’s syndrome, and multiple sclerosis) intestinal dysbiosis has been found^[98]^. Further studies are needed to understand whether the intestinal microbiota may be one of the triggers that initiate the autoimmune process in predisposed individuals and contribute to the onset of the disease. Moreover, it is necessary to understand if modulation with pro-and prebiotics can be helpful to prevent or slow down this process.

Finally, studies have explored whether variation in the composition of the gut microbiota may have an impact on thyroid cancer and benign nodules^[99-101]^. In this context, it was observed that people with high-grade thyroid nodules have significant alterations in the overall microbial composition, showing fewer intestinal microbial species and functionally fewer gene families responsible for amino acid degradation and butyrate production, compared to those with lower grade nodules^[101]^. Furthermore, various microRNAs appear to regulate the signaling of thyroid hormones in tissues. In turn, thyroid hormones modulate the expression of specific miRNAs and their mRNA targets in different types of cells and organs^[102]^ and seem to also be involved in cell proliferation and cancer^[103]^.

In conclusion, the relationship between thyroid function, thyroid diseases, specific pharmacological therapies on the one hand and variations in the microbiota on the other hand appears complex and is still not well defined. However, various pre-clinical and clinical evidence confirms that this relationship exists and must lead us to address this topic in a systematic way and with more consolidated and updated methodologies.

## CONCLUSIONS

Inter-kingdom cell-to-cell signaling, involving small molecules, i.e., hormones produced by eukaryotes and hormone-like chemicals produced from bacteria, is found among mammals and in plant-bacteria relationships and turns out to be a fascinating field of research for its implication on the physiology of the host. In this context, it is widely accepted by the scientific community that the human microbiota, and in particular the gut microbiota, has a profound impact on human health. Thus, all the possible effector molecules, including small molecules produced by the human body that might modify the gut microbiota composition, could influence the host’s health status. Notably, data are accumulating that show specific alterations in hormone levels might be responsible for modifying the the composition and functionality of the infant gut microbiota. Such a phenomenon is even more important in the pediatric age, where the gut microbiota is more susceptible to modifications influenced by breast milk and the mother’s health. Furthermore, it appears that several mom-related factors also play a significant role in the infant microbiota establishment, i.e., gestational age, mode of delivery, type of feeding, antibiotic exposure, host genetics, environment, and diet, as well as an intricate relationship which involves the immune, nervous, and endocrine systems. In this context, a new avenue of research is opening up on the understanding of the roles exploited by maternal hormones carried during pregnancy or later by human milk on the infant gut microbiome and, ultimately, on the baby’s health status. 

## DECLARATIONS

### Copyright

© The Author(s) 2022.
